# Implications of year 1 Trump Administration policies for Americans’ lung health

**DOI:** 10.1093/ajrccm/aamag095

**Published:** 2026-03-03

**Authors:** Adam W Gaffney, Steffie Woolhandler, David C Christiani, Mary B Rice, John Balmes, C Corey Hardin, Cecile Rose, David Himmelstein, Philip J Landrigan, The Lung Health Policy Tracking Group, Adam W Gaffney, Steffie Woolhandler, David C Christiani, Mary B Rice, John Balmes, C Corey Hardin, Cecile Rose, David Himmelstein, Philip J Landrigan

**Affiliations:** Harvard Medical School, Boston, MA, United States; Department of Medicine, Cambridge Health Alliance, Cambridge, MA, United States; Harvard Medical School, Boston, MA, United States; Department of Medicine, Cambridge Health Alliance, Cambridge, MA, United States; City University of New York, Hunter College, New York, NY, United States; Harvard Medical School, Boston, MA, United States; Harvard T.H. Chan School of Public Health, United States; Department of Medicine, Massachusetts General Hospital, Boston, MA, United States; Harvard Medical School, Boston, MA, United States; Harvard T.H. Chan School of Public Health, United States; Department of Medicine, Beth Israel Deaconess Medical Center, Boston, MA, United States; Department of Medicine, University of California San Francisco, San Francisco, CA, United States; School of Public Health, University of California Berkeley, Berkeley, CA, United States; Harvard Medical School, Boston, MA, United States; Department of Medicine, Massachusetts General Hospital, Boston, MA, United States; Department of Medicine, National Jewish Health, Denver, CO, United States; Department of Medicine, University of Colorado Anschutz Medical Campus, Aurora, CO, United States; Harvard Medical School, Boston, MA, United States; Department of Medicine, Cambridge Health Alliance, Cambridge, MA, United States; City University of New York, Hunter College, New York, NY, United States; Global Observatory on Planetary Health, Boston College, Chestnut Hill, MA, United States; Centre Scientifique de Monaco, Monaco, MC, United States

In 2025, the Trump Administration undertook multiple actions with major ramifications for the pulmonary health of Americans.

The “One Big Beautiful Bill Act” (OBBBA), signed into law by President Trump on July 4, 2025, will terminate healthcare coverage for many low-income Americans with lung disease. The administration’s rollback of environmental protections and reduction in subsidies for clean energy production will worsen air quality and accelerate climate change. Cuts at the Centers for Disease Control and Prevention (CDC) could disrupt occupational health protections and tobacco and asthma control efforts; the Department of Health and Human Services (HHS) leadership’s embrace of anti-vaccine theories will drive vaccine-preventable illness. The termination of federally funded research grants, including some focused on respiratory diseases, could slow medical progress. And sweeping disruptions to global health aid may impede global tuberculosis control.

In this article, we assess the likely impacts on Americans’ pulmonary health, across the life course, of policies proposed or enacted in the first year of the second Trump Administration, drawing on peer-reviewed studies, media reporting, Congressional Budget Office (CBO) reports, government agencies’ impact analyses, and our analyses of national survey data.

## Healthcare for patients with lung disease

The OBBBA will have sweeping effects on Americans’ healthcare coverage, particularly Medicaid, the public insurance program that covers more than 70 million low-income Americans. It will: (1) require about 20 million Medicaid enrollees to document work hours or exemptions, or lose coverage; (2) roll back streamlined Medicaid enrollment procedures and impose more frequent eligibility re-determinations; (3) impede enrollment in subsidized ACA marketplace plans; and (4) constrain states’ use of provider taxes, which help finance their Medicaid programs, among many other provisions.^1^ The CBO projects the law will cut federal Medicaid and ACA spending by nearly $1 trillion over a decade,^2^ and add 10 million low-income individuals to the ranks of the uninsured.^3^ These healthcare cuts were imposed to partially offset the cost of the OBBBA’s tax cuts (which mostly benefit the wealthy), and a tripling of spending on immigration enforcement.

**Figure 1:**
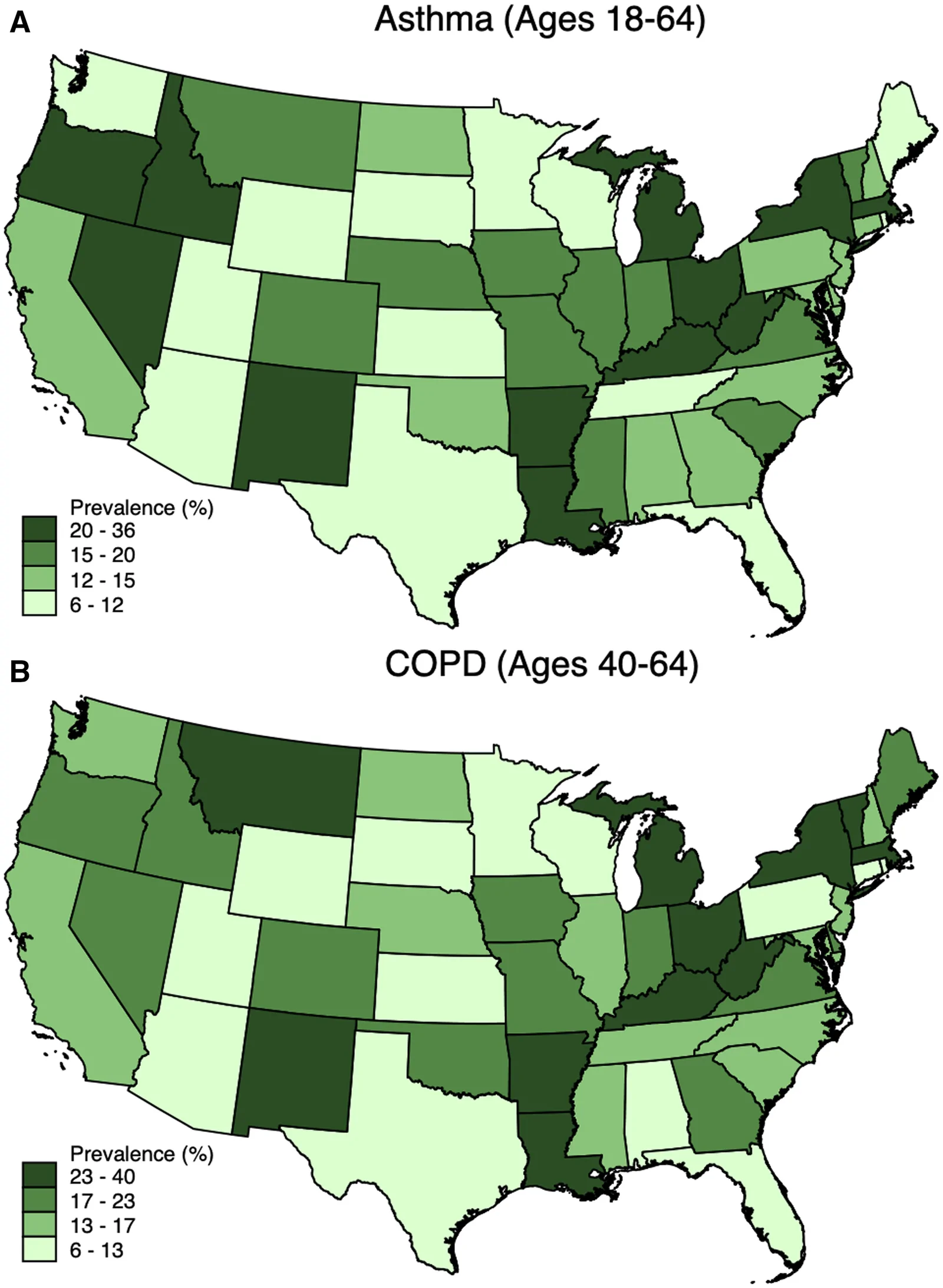
Percentage of non-elderly adults with asthma (ages 18-64) or COPD (ages 40-64) with Medicaid as primary health coverage, 2022-2024. Based on authors’ analysis of 2022-2024 Behavioral Risk Factor Surveillance System (BRFSS) microdata (*n* = 832 629 person-level records analyzed of individuals under 65). Hawaii and Alaska not shown for presentation purposes; the share of non-elderly patients with asthma who had Medicaid as primary insurance was 7.8% and 21.5% in these 2 states, respectively; the corresponding shares of those with COPD with Medicaid as primary insurance was 9.4% and 24.6%. Data on territories not shown given differential policy implications.

Many of those left uninsured will be individuals with chronic lung disease. Our analysis of data from the 2022-2024 Behavioral Risk Factor Surveillance System (BRFSS) indicates that 3.2 million adults (ages 18-64) with asthma and 1.3 million ages 40-64 with chronic obstructive pulmonary disease (COPD) have Medicaid as their primary insurance coverage. Large shares of these patients in both “red states” (eg, ≥30% of asthma patients in West Virginia and Louisiana) and “blue states” (eg, 40% of COPD patients in Washington, D.C.) rely primarily on Medicaid (Figure 1).

Loss of coverage will interrupt primary and pulmonary specialty care for many such patients^4^^,^^5^; lead to discontinuation of medications that control or slow disease progression (including controller inhalers,^4^^,^^6^ biologic therapies,^7^ immunosuppressives, antifibrotics, and oncologic drugs); reduce use of tobacco cessation treatment^8^; lower rates of vaccination against respiratory pathogens^9^; reduce participation in pulmonary rehabilitation; diminish access to supplemental oxygen for patients with chronic hypoxia; delay patients’ access to emergency care during disease exacerbations; and impede lung transplantation.^10^ The OBBBA also imposes copays (up to $35) for specialty care on many Medicaid beneficiaries, which may deter pulmonary outpatient care even for those who retain coverage.

**Figure A1:**
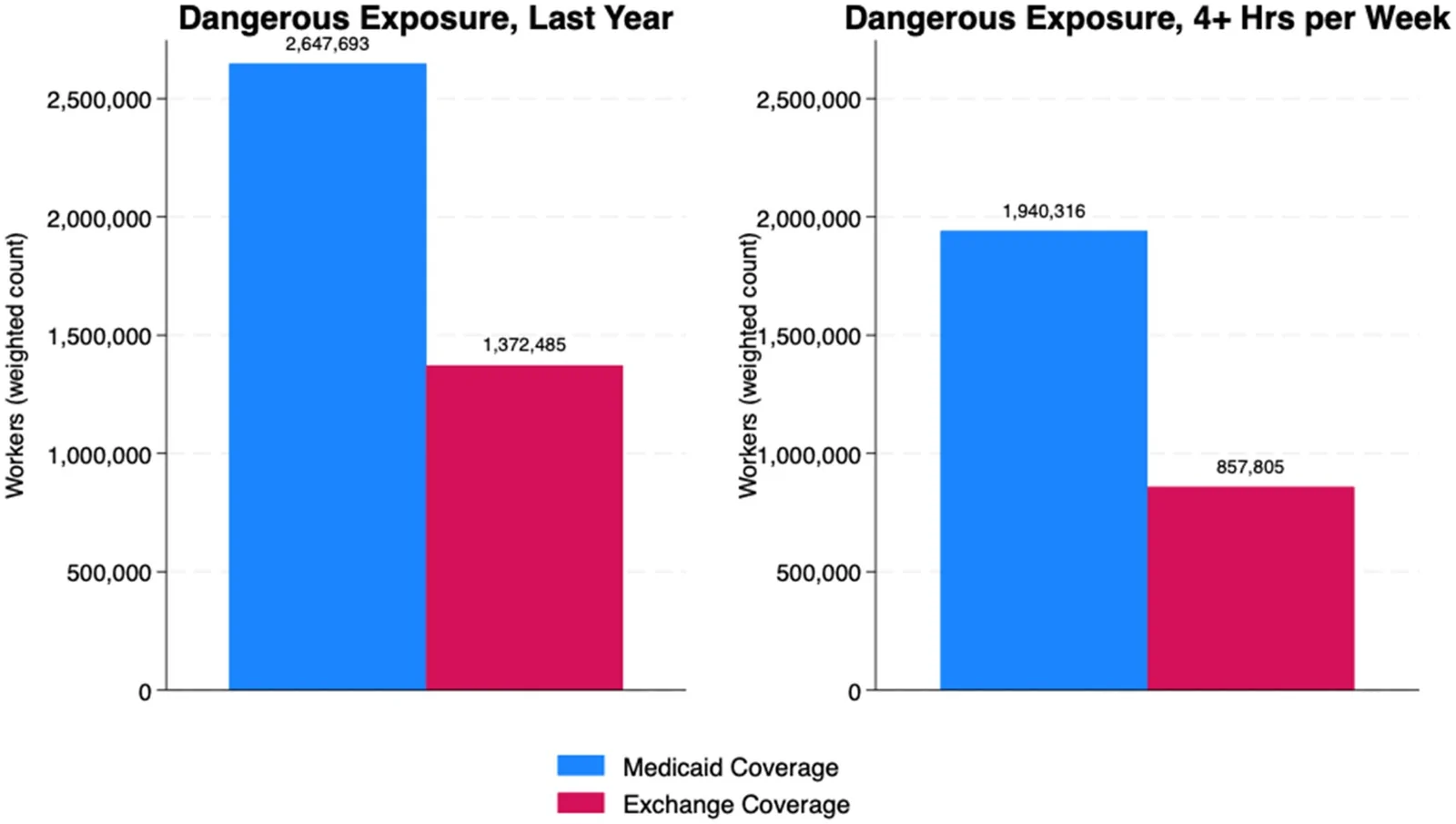
Estimates of Medicaid and ACA marketplace coverage among Non-Elderly adult workers with reported exposure to dangerous substances in the workplace in the United States, 2023. Our analysis of National Health Interview Survey (NHIS) data below shows that among workers reporting occupational exposures to dangerous substances, 2.6 million were covered by Medicaid and 1.4 million by exchange plans in 2023. Proposed cuts to Medicaid and ACA may lead to shifting of these workers’ healthcare claims costs to workers’ compensation carriers, resulting in delays and denials in access to care. Contingent, contract, and low-wage workers are most likely to be affected by these coverage losses and eligibility hurdles. Estimates are from our analysis of the 2023 National Health Interview Survey (NHIS), obtained via IPUMS. We analyzed data on *n* = 2361 adults ages 18-64 with an affirmative response to the question, “During the past 12 months, did you work in a job where you were exposed to any of the following: solvents, industrial glues, heavy metals, pesticides, or motor engine exhaust?” If necessary, the following clarification text was read: “Solvents are liquids, not including water, used for dissolving other substances, such as dry-cleaning fluids, thinners, components of paints, industrial cleaners, and paint removers. Industrial glues are adhesives that include acrylic, epoxy, hot melt, polyurethane, silicone, thermoset, and industrial sealants. Heavy metals include lead, nickel, mercury, cadmium, arsenic, chromium, zinc, and copper.” Those with an affirmative response were then asked if they were exposed to such substances for 4 or more hours per week. We calculated national estimates for counts of such workers with Medicaid and Exchange coverage using NHIS’ survey weights.

Medicaid and ACA cuts could also lead to delayed care for occupational injuries and illnesses among workers in construction, mining, and agriculture, increasing the risk of chronic disability and premature death among sick and injured workers (Figure A1).

Additionally, a sharp reduction in Medicaid funding will likely undermine the finances of hospitals that care for publicly insured, low-income populations,^11^ leading to cuts in services to both Medicaid and non-Medicaid patients, and possibly even closure of some hospitals (particularly in rural areas). Such hospital impacts could exacerbate current shortfalls and disparities in pulmonary care and ICU infrastructure^12^ highlighted by the COVID-19 pandemic.^13^

Finally, while the administration has called attention to the problem of high US drug prices, and negotiated deals for lower prices with some drug firms for a small number of drugs, drug firms announced price increases on 872 drugs in 2026, more than the previous year.^14^

## Environmental health

In keeping with President Trump’s campaign promise to roll-back environmental protections, the Environmental Protection Agency (EPA) Administrator announced the “biggest deregulatory action in US History” in March 2025.^15^ Many of the 31 proposed deregulatory actions will, directly or indirectly, worsen air quality, including: weakening the health-based standards for fine particulate matter (PM2.5) pollution^16^ under the National Ambient Air Quality Standards (NAAQS) program from 9 µg/m^3^ up to 12 µg/m^3^; delaying (and possibly repealing) regulations that mitigate methane (and other) emissions from oil and gas extraction^17^; weakening protections against particularly toxic “hazardous air pollutants” like mercury^18^^,^^19^; ending Biden-era rules limiting carbon emissions from coal-fired power plants^20^; relaxing standards for motor vehicle fuel efficiency and tailpipe emissions^21^^,^^22^; and abandoning the EPA’s “Endangerment Finding” that permits regulation of greenhouse gas emissions as pollutants.^23^ The Trump Administration has also blocked or attempted to block clean energy projects (including a large wind farm in Rhode Island that was near completion^24^^,^^25^); terminated green energy and electric vehicle subsidies provided by the Inflation Reduction Act^25^; mandated continued operation of aging fossil-fueled power plants that had been set for closure,^26^ and proposed expansion of offshore drilling for oil in public waters off the coasts of California, Florida, and Alaska.^27^ The administration additionally prompted Congress to rescind California’s waiver under the Clean Air Act that mandated purchases of zero-emission vehicles, a policy followed by 12 other states.^28^

These rollbacks could benefit some polluting corporations and industries. However, they will deprive Americans—including young children with developing lungs and people living with chronic lung disease—of health-based protections. Many of the respiratory health consequences of these actions are likely to be irreversible, as air pollution exposure causes permanent deficits in lung development, worsens asthma and COPD, and contributes to premature death. Previous EPA analyses of 5 rules likely to be rescinded (Table 1) projected that these rules would prevent thousands of new cases of asthma, hundreds of thousands of asthma exacerbations, thousands of respiratory emergency department visits, hundreds of respiratory illness hospitalizations, and many deaths. Rescinding these policies will turn these potential benefits into harms for respiratory health.

**Table 1:** Potential pulmonary health impacts of 5 proposed De-Regulatory actions based on environmental protection agency (EPA)’s previous analyses of their expected annual benefits.

	Change in annual asthma incidence	Change in annual incidence of asthma symptoms among children	Change in annual respiratory ED visits	Change in annual respiratory hospital admissions	Year of EPA projection	Reference number
**1. Rollback primary PM2.5 standard from 12 to 9 mcg/m3**	5700	800 000	1300	230	2032	^92^
**2. Rollback pollution standards for the Oil and Gas Industry**	520	97 000	170	9	2038	^93^
**3. Rollback carbon standards for fossil-fuel power plants**	1910	360 000	560	55	2035	^94^
**4. Rollback car and truck tailpipe emissions standards**	5800	1 090 000	1860	164	2055	^95^
**5. Rollback wastewater standards for coal-power plants**	600	114 000	196	18	2035	^96^

ED = emergency department. When estimates were available for more than 1 year, 2035 estimates (or closest year) were selected; year of estimates (and source) shown in final column. Estimates are point estimates for each policy; impacts from PM2.5 and Ozone were summed when separately provided. Estimates were rounded to nearest integer. Policy impacts of these provisions may overlap and estimates should not be assumed to be cumulative. See sources for details on methodology. For policy 1, asthma symptoms defined as exacerbated asthma with albuterol inhaler use among children 6-13 (Table 5-2; Table ES-6^92^); for policy 2, asthma symptoms defined as “asthma symptoms/exacerbation” among asthmatics age 5-17 (Table 3-1; Table 3-9^93^); for policy 3, asthma symptoms defined as “asthma symptoms/exacerbation” for those ages 6-17 for PM2.5 impacts and “asthma symptoms/exacerbation” among asthmatics ages 2-17 for ozone impacts (Table 4-7 and Table 4-12^94^); for policy 4, as “exacerbated asthma-albuterol inhaler use” among asthmatics ages 6-13 for PM2.5 but among those aged 5-17 for ozone (Tables 7-16, 7-17, 7-18^95^); and for Policy 5 as “asthma symptoms/exacerbation” among those ages 6-17 for PM2.5 but among those ages 2-17 for ozone (Tables 8-9, 8-10^96^).

Over the longer term, these rollbacks will also increase climate-forcing carbon emissions that accelerate global warming and worsen respiratory health in the United States and globally by increasing exposures to extreme heat, wildfire smoke, allergens, floods, and other adverse climate events.

## Occupational lung disease

### NIOSH staff and funding

In 1970, Congress established the National Institute for Occupational Science and Health (NIOSH) to conduct the scientific studies needed to advance occupational health and safety protections. In April 2025, the Trump Administration fired most NIOSH staff, putting key programs responsible for occupational lung disease prevention (eg, the Coal Workers’ Health Surveillance Program and the Respirator Certification Program), as well as training programs for occupational health professionals, at risk.^29-31^

However, in the face of lawsuits, protests, and persistent political pressure from organized labor and others, some NIOSH staff were rehired in 2025, and in January 2026, the cuts were fully reversed.^30^^,^^32^^,^^33^ And although the Trump Administration’s budget proposal called for a 20% reduction in NIOSH funding,^34^ this cut was rejected by Congress, which passed a budget bill in February 2026 that restored NIOSH funding.

### Workplace regulations

Silica exposure puts workers in several industries at risk for fatal lung diseases including silicosis and lung cancer. To counter rising silica-related deaths, pulmonologists and other health professionals advocated for decades for better workplace protections. In 2016, the Occupational Safety and Health Administration (OSHA) issued a new silica rule that increased protections for workers in the construction, “general industry,” and maritime sectors. The Mine Safety and Health Administration (MSHA), responsible for promulgating standards in mines, finalized similar protections in 2024, to be enacted in June 2025.

However, in April 2025, MSHA delayed implementation of the new silica rule until August, citing disruptions at NIOSH, and further delayed implementation in response to litigation filed by an industry group. Meanwhile, Congressional Republicans have called for the abandonment of the silica rule altogether,^35^ and in December, the administration stated that it would “reconsider” at least parts of it.^36^ The fate of the rule—projected to prevent more than 1000 silicosis deaths over 6 decades^37^—is unclear.

Other Administration actions could increase miners’ risk of occupational lung disease. District managers at the MSHA are responsible for reviewing health and safety plans devised by mine companies, and can request specific additional protections on a case-by-case basis, including requiring heightened ventilation standards.^38^ In August, the Department of Labor announced plans to revoke managers’ authority to make such requests.^38^

## Immigrant health

Many non-citizen immigrants have chronic lung disease and rely on public insurance for coverage (Table A1). Several policies advanced by the Trump administration may endanger their care and health. For instance, in addition to the provisions noted above, the OBBBA will end eligibility for publicly subsidized healthcare for some categories of legally present immigrants, including refugees, asylees, and certain victims of trafficking and abuse.^39^ The OBBBA also reduces funding for Emergency Medicaid, which covers the costs of emergency care for some immigrants. Meanwhile, a rule finalized by the Trump Administration will reverse DACA recipients’ eligibility for ACA marketplace coverage.^39^ While the administration has defended such measures as cost-saving, immigrants’ contributions to public and private health insurance programs exceed their care costs.^40^

The administration’s anti-immigrant rhetoric and actions, including militarized raids by Immigration and Customs Enforcement (ICE), are also expected to reduce immigrants’ participation in public programs like Medicaid, even among those still eligible,^41^^,^^42^ through well-described “chilling effects”^43^ that deter immigrants (including those with lung disease) from obtaining coverage or care. The Administration has also provided non-citizen Medicaid data to ICE for potential use in immigration enforcement, and has withdrawn traditional protections against immigration enforcement activities in “sensitive” locations like hospitals—actions likely to augment such “chilling” effects.^39^

Escalating use of chemical irritants by immigration enforcement agents during protests,^44^ moreover, imposes immediate threats to individuals with lung disease,^45^ with reported exposure of 2 children with severe asthma in Minneapolis.^46^

Finally, since more than 1 million in the US healthcare workforce are non-citizen immigrants who provide essential care services,^47^ repressive enforcement actions could undercut care provision in hospitals, long-term care facilities, and other settings—worsening care for patients with lung disease and the critically ill, regardless of immigration status. Similarly, immigrants have been responsible for major scientific advances; escalating restrictions and repressive enforcement, including against scientists and students, may undercut biomedical research.

## Other public health programs

On April 1, the Trump Administration fired some 10 000 HHS staff, including many at CDC.^48^ Along with the NIOSH cutbacks noted above, two other CDC divisions focused on pulmonary health—the National Asthma Control Program (NACP) and the Office of Smoking and Health (OSH)—experienced sharp reductions in funding and staff, which media reports characterized as their near elimination.^49^^,^^50^

The NACP, established in 1999, has conducted surveillance and epidemiologic research on asthma, and funded and helped implement evidence-based asthma control programs in collaboration with state and local health departments.^51^ Its reduction or dissolution would undermine efforts to mitigate asthma morbidity and mortality.^52^ The Office of Smoking and Health (OSH), established in 1978,^53^ has conducted tobacco use surveillance, funded and helped implement smoking cessation programs, and carried out national campaigns communicating the hazards of cigarettes that have been credited with large declines in cigarette use and mortality.^49^^,^^53^^,^^54^ Staffing losses at OSH could undermine such tobacco control efforts; indeed, disruptions in federally funded smoking cessation programs were already reported as a consequence of cuts in 2025.^49^^,^^55^

However, following the Department of Government Efficiency (DOGE)-led firings in April 2025, legal challenges and political pressure led to the rehiring of many HHS workers, although litigation left many in limbo.^56^ And while Trump’s budget called for a dramatic 25% reduction in HHS funding^57^ and the elimination of NACP,^52^ these cuts were rejected by Congress, which in February 2026 passed a budget bill that kept CDC funding effectively flat,^58^ and that restored divisions including the NACP.^59^

However, other administration policies could undercut tobacco control efforts. The FDA’s Center for Tobacco Products (CTP), which regulates cigarette sales and marketing,^60^ also experienced staff reductions in 2025. Such cuts could undermine progress on the FDA’s nicotine reduction rule,^61^ introduced in the final days of the Biden Administration, which the FDA has estimated would save 1.8 million lives over 4 decades.^62^ Staffing aside, it remains unclear whether the Trump Administration will ultimately choose to advance such a regulation,^61^ which was absent from the FDA’s regulatory agenda published in September 2025, and faces stiff opposition from the tobacco industry.^63^ Finally, in January 2025, the Trump Administration terminated a menthol cigarette ban, also opposed by industry, that the Biden Administration had proposed but never implemented.

## Vaccine science and uptake

Vaccines against influenza, respiratory syncytial virus (RSV), COVID-19, pertussis, and pneumococcus are especially important for patients with chronic lung disease. The health coverage losses described above will likely reduce uptake of these vaccines among these patients. Other administration initiatives and rhetoric could further impede vaccine uptake. Kennedy (with Trump) has promulgated vaccine misinformation, particularly claiming a link between childhood vaccinations and autism; emphasized treatment (eg, with cod liver oil) over vaccination in the face of a national measles outbreak^64^; and dismissed all members of the CDC’s Advisory Committee on Immunization Practices (ACIP), who were replaced with a panel that includes many vaccine skeptics.^65^

There have been other major shifts in vaccine policy, including those targeting respiratory pathogens. In February 2025, the CDC prematurely ended its “Wild to Mild” influenza vaccination outreach campaign amidst the worst flu season in more than a decade.^66^ The FDA also narrowed authorization of COVID vaccines to higher risk populations, deterring access for some patients. And in January 2026, the CDC—following an order from Trump—abruptly upended the entire pediatric vaccine schedule; RSV vaccines were recommended only for high-risk children, and influenza and COVID-19 only on the basis of “shared clinical decision making.”^67^

Finally, HHS’ cancellation of $500 million in grants for mRNA vaccine development could slow development of new vaccines in the face of future viral respiratory epidemics.

## International aid and global health

Through a series of executive actions, President Trump upended US global health activities in 2025,^68^ including withdrawing from the World Health Organization (WHO), a stop-work order, the dissolution of the United States Agency for International Development (USAID), firings at the CDC, and disruptions in funding for PEPFAR (a leading funder of AIDS prevention and treatment programs in low-income nations).^69^

Disruptions in funding of programs for tuberculous (TB) detection, prevention, and treatment could have the greatest impact on lung health. One modeling study projected that such funding cuts could lead to between 99 900 and 2.2 million TB deaths worldwide^70^; another found that termination of USAID funding alone could result in an additional 1.4 million cases of TB and more than 500 000 deaths.^71^ Deterioration of global TB control could also increase TB transmission into the United States, including of multi-drug resistant strains. Meanwhile, while a waiver theoretically exempted PEPFAR from the administration’s global health cuts, the Administration withheld billions of dollars in PEPFAR funding, at least temporarily disrupting program activities^69^ and likely increasing HIV transmission and deaths as well as opportunistic pulmonary infections like TB. A modeling study projected that a 50% reduction in PEPFAR funding for South Africa alone would lead to 315 000 deaths over 10 years.^72^

However, as in other areas, in early 2026 Congress rejected the dramatic global health cuts called for in the Trump Administration’s budget for the coming fiscal year. How the Administration will use allocated funds, and the impacts of its new “American First Global Health Strategy”—focused on bilateral agreements (including demands for access to mineral deposits) with low-income nations—raise concerns about the future of US global health aid.^73-76^

## Research funding

In 2025, the Trump Administration significantly disrupted biomedical research through funding cuts to the National Institutes of Health (NIH) and National Science Foundation (NSF). In addition to the abrupt cancellation of thousands of research grants, it imposed funding blockades on specific targeted universities. Defunding of research on topics such as health inequities, social determinants of lung health,^77^ vaccine hesitancy, COVID-19, and COPD exacerbation prevention^78^ could have long-term adverse impacts on pulmonary patients’ health.^77^ These policies also disrupted more than 380 NIH-funded clinical trials, including at least 7 focused on respiratory disease.^79^ Even as legal action reversed some or most of these grant cancellations, interruption of clinical trials can result in irreparable damage to these studies.

Trump’s budget called for a 40% reduction in NIH funding for 2026. Yet again, Congress rejected these cuts, passing a budget bill in February 2026 that provided a small increase in NIH funding.^80^ However, political pressure to avoid important research priorities—including reduction of disparities in lung health—will likely endure.^77^

## Women’s health

The first year of the Trump Administration saw a further degradation in access to reproductive healthcare, including abortion care, already severely undermined by the 2022 *Dobbs* decision. The administration reinstated the Mexico City Policy, cutting aid to foreign organizations that provide any abortion care^81^; imposed aforementioned Medicaid cuts and consequent coverage losses that will heavily effect reproductive-age women; eliminated Medicaid reimbursement for all services at Planned Parenthood; and launched (but then delayed^82^) a “safety review” of mifepristone (used for medication abortion) despite the drug’s long-track record of safety. For individuals with severe respiratory disease, such as pulmonary hypertension (which predominantly affects reproductive-age women), such restrictions could impede access to therapeutic abortion with potentially dire (even lethal) health consequences.^83^

## Pediatric lung health

The respiratory health of children will be harmed by policies in nearly all the aforementioned categories. For example, while the largest Medicaid cut (Work Requirements) targets adults, other cuts (eg, policies that increase enrollment barriers) could inflict coverage losses on children, including those with asthma. Pollution rollbacks, meanwhile, will harm the developing lung in utero and in early life, causing lifelong decrements in respiratory function^84^ and increased risk of asthma—likely worsening racial and socioeconomic disparities in respiratory health that emerge^85^ in early-life. Measles epidemics stemming from deteriorating vaccination rates will disproportionately endanger children, who risk severe complications including pneumonia and resultant long-term pulmonary sequelae; increases in other vaccine-preventable respiratory tract infections could also impede children’s long-term lung development.^86^ Children will also bear the brunt of the long-term health harms stemming from climate change.

Other policies could impose additional harms on children’s respiratory health. For instance, nutrition is an important determinant of healthy lung development^87^; while the administration has called attention to the problem of poor childhood nutrition, the OBBBA’s $200 billion cut to the Supplemental Nutritional Assistant Program (SNAP), which provides food assistance to low-income families, could increase maternal and early childhood food insecurity, potentially harming lung health and development.

## Discussion

Lung disease is a leading cause of suffering and death. Policies pursued or enacted during the Trump Administration’s first year will likely increase pulmonary morbidity and mortality, and widen already growing^88^ inequalities in lung health and care. These policies—a form of pulmonary structural violence^89^—strip away essential measures that protect the respiratory health of all Americans, from infancy through old age: they appear set to degrade patient care, worsen air quality, accelerate climate change, increase vaccine-preventable and exposure-related respiratory illnesses, undermine tobacco control, upend global TB control, and hobble scientific progress on lung disease. Such harms—particularly to the developing lungs of children—will long outlast the Trump Administration. In contrast, corporations facing relaxed pollution standards stand to benefit, as do wealthy individuals who are unlikely to work in dangerous jobs or live in polluted areas and who are the primary beneficiaries of tax cuts.

The pulmonary and critical care medicine community—including our already outspoken professional societies^90^—should respond vigorously to this crisis. We must amplify the urgent message that these actions are inflicting imminent harms on our patients. Research should track these policies’ impacts and the magnitude of their harms. Finally, we should advocate for alternative policies informed by decades of research to not only restore, but improve and expand care for every patient with respiratory disease,^91^ safeguard workers’ lungs, fight tobacco use, and ensure clean air for all.

## Supplementary Material

aamag095_Supplementary_Data

## Data Availability

The data analyzed to produce Figure 1 is publicly available at: https://www.cdc.gov/brfss/annual_data/annual_data.htm. The data analyzed to produce Appendix Figure A1 and Table 1 is publicly available at: Lynn A. Blewett, Julia A. Rivera Drew, Andrew Fenelon, Miriam L. King, Kari C.W. Williams, Daniel Backman, Etienne Breton, Grace Cooper, and Stephanie Richards. IPUMS Health Surveys: National Health Interview Survey, Version 8.1 [dataset]. Minneapolis, MN: IPUMS, 2025. https://doi.org/10.18128/D071.V8.1. There are otherwise no new data associated with this article. This article has an online data supplement, which is accessible at the Supplements tab.

## Appendix

**Table A1:** Estimates of Medicaid and ACA marketplace coverage among non-citizen immigrants with asthma and chronic obstructive pulmonary disease (COPD), 2020-2024.

	Asthma	COPD
**Total non-citizens, any coverage**	638 267	249 241
**Total non-citizens, Medicaid**	154 176	79 088
**Total non-citizens, exchange coverage**	50 836	12 321

Estimates are from our analysis of the 2020-2024 National Health Interview Survey (NHIS), obtained via IPUMS; we analyzed data on *n* = 10 373 non-US citizens. Following usual practice, weights were divided by the number of survey.
