# Supplementary material for: Implications of year 1 Trump Administration policies for Americans’ lung health
Source: Am J Respir Crit Care Med. 2026 Mar 3;212(5):900–10. doi: 10.1093/ajrccm/aamag095 (PMC13160938; doi:10.1093/ajrccm/aamag095)
Supplement: Supplementary file 1 [file aamag095_supplementary_data.zip › aamag095_Supplementary_Data/Appendix R1.docx]

**Appendix Figure 1: Estimates of Medicaid and ACA Marketplace Coverage Among Non-Elderly Adult Workers with Reported Exposure to Dangerous Substances in the Workplace in the United States, 2023**

Our analysis of National Health Interview Survey (NHIS) data below shows that among workers reporting occupational exposures to dangerous substances, 2.6 million were covered by Medicaid and 1.4 million by exchange plans in 2023. Proposed cuts to Medicaid and ACA may lead to shifting of these workers’ healthcare claims costs to workers’ compensation carriers, resulting in delays and denials in access to care. Contingent, contract and low-wage workers are most likely to be affected by these coverage losses and eligibility hurdles.

Notes: Estimates are from our analysis of the 2023 National Health Interview Survey (NHIS), obtained via IPUMS. We analyzed data on n=2,361 adults ages 18-64 with an affirmative response to the question, “During the past 12 months, did you work in a job where you were exposed to any of the following: solvents, industrial glues, heavy metals, pesticides or motor engine exhaust?” If necessary, the following clarification text was read: “Solvents are liquids, not including water, used for dissolving other substances, such as dry-cleaning fluids, thinners, components of paints, industrial cleaners and paint removers. Industrial glues are adhesives that include acrylic, epoxy, hot melt, polyurethane, silicone, thermoset and industrial sealants. Heavy metals include lead, nickel, mercury, cadmium, arsenic, chromium, zinc and copper.” Those with an affirmative response were then asked if they were exposed to such substances for 4 or more hours per week. We calculated national estimates for counts of such workers with Medicaid and Exchange coverage using NHIS’ survey weights.

**Appendix Table 1: Estimates of Medicaid and ACA Marketplace Coverage among Non-Citizen Immigrants with Asthma and Chronic Obstructive Pulmonary Disease (COPD), 2020-2024**

|  | Asthma | COPD |
| --- | --- | --- |
| Total non-citizens, any coverage | 638,267 | 249,241 |
| Total non-citizens, Medicaid | 154,176 | 79,088 |
| Total non-citizens, Exchange Coverage | 50,836 | 12,321 |

Notes: Estimates are from our analysis of the 2020-2024 National Health Interview Survey (NHIS), obtained via IPUMS; we analyzed data on n=10,373 non-US citizens. Following usual practice, weights were divided by the number of survey
