# Supplementary material for: Implications of year 1 Trump Administration policies for Americans’ lung health
Source: Am J Respir Crit Care Med. 2026 Mar 3;212(5):900–10. doi: 10.1093/ajrccm/aamag095 (PMC13160938; doi:10.1093/ajrccm/aamag095)
Supplement: Supplementary file 1 [file aamag095_supplementary_data.zip › aamag095_Supplementary_Data/ODS title page.docx]

**ONLINE DATA SUPPLEMENT**
